# Pulsed thermographic analysis of Herculaneum papyri

**DOI:** 10.1038/s41598-025-19911-w

**Published:** 2025-10-08

**Authors:** Sofia Ceccarelli, Massimo Rippa, Giovanni Caruso, Loredana Luvidi, Simona Boccuti, Melania Paturzo, Vito Pagliarulo, Kilian Fleischer, Costanza Miliani, Graziano Ranocchia

**Affiliations:** 1 Institute of Heritage Science (CNR-ISPC), National Council of Research, Naples, Italy; 2 Institute of Applied Sciences and Intelligent Systems ‘E. Caianiello’ (CNR-ISASI), National Council of Research, Pozzuoli, Italy; 3 Institute of Classical Philology, Faculty of Philosophy, University of Tübingen, Tübingen, Germany; 4 Department of Philology, Literature and Linguistics, University of Pisa, Pisa, Italy

**Keywords:** Imaging techniques, Archaeology, Characterization and analytical techniques

## Abstract

The Herculaneum papyri represent an exceptional cultural treasure, providing invaluable insights into the philosophical, literary and historical landscape of the Greco-Roman world. However, their carbonization due to the eruption of Mount Vesuvius in 79 AD poses significant challenges for both textual legibility and structural analysis. This study illustrates the first application of Pulsed Thermography to the non-destructive analysis of these unique manuscripts. By employing an experimental setup comprising a couple of flashes providing the excitation and a thermal camera for recording the infrared emission from the sample, the technique proves to be an important tool for textual recovery, revealing writing almost not discernible to the naked eye. Furthermore, Pulsed Thermography provides structural information useful for restorers, such as the papyrus texture and adhesion points to paperboard, offering a valuable advantage over other imaging techniques. The results show the potential of the adopted method not only as a powerful tool for textual recovery but also for guiding conservation strategies ensuring a deeper understanding of Herculaneum papyri.

**Supplementary Information:**

The online version contains supplementary material available at 10.1038/s41598-025-19911-w.

## Introduction

The Herculaneum papyri, kept in the Officina dei Papiri Ercolanesi of the National Library ‘Vittorio Emanuele III’ of Naples, represent one of the most extraordinary archaeological discoveries of all times and the only library to have come down to us directly from antiquity^1,2^. These precious manuscripts survived from the catastrophic eruption of Mount Vesuvius in 79 AD thanks to the particular thermal interaction between the pyroclastic deposits and the edifice where they were stored^3,4^. The thermal shock caused by the high temperatures (ca. 320 °C) of the volcanic surge that buried Herculaneum allowed the preservation by carbonization, at controlled atmosphere and in dehydration conditions, of the papyrus scrolls. These were buried under more than 30 m of volcanic material and were discovered in the mid-18th century in the Villa dei Papiri, a magnificent suburban estate believed to have belonged to Julius Caesar’s father-in-law Lucius Calpurnius Piso Caesoninus^5–7^. Most of these scrolls were unrolled through the Piaggio machine, a mechanical unrolling system designed by the Genoese Father Antonio Piaggio^2,8^. They contain unknown Greek philosophical texts, many of which are attributed to the Epicurean philosopher Philodemus of Gadara (110-after 40 BC) so being an invaluable source for historians of ancient philosophy and literature^1,9^. These papyri represent a unique challenge for modern science and technology owing to their carbonization, layering, and hard legibility, so requiring innovative approaches to their study and preservation^10^. Moreover, like most of ancient papyri, Herculaneum papyri are written with carbon-based ink, which has an elemental composition similar to that of the papyrus substrate, although low traces of metals in macroscopic ink marks on some small fragments were recently detected^11–13^. Given these challenges, the opportunity of employing non-invasive advanced techniques for their study has become increasingly impelling over time. In the last twenty-five years, the introduction of various techniques has led to unexpected results, such as the successful reading of unrolled papyri through near-infrared technical photography^14^, the partial virtual unrolling and deciphering of unopened papyri by using x-ray computed-tomography techniques^15–17^ and the revealing of portions of Greek text hidden on the back of an opisthograph roll by using shortwave-infrared hyperspectral imaging^18^. More recently, the application of macro-x-ray fluorescence imaging provided the first experimental proof of the use by ancient scribes of lead-drawn ruling lines for the layout of Greek papyrus bookrolls^19^. These studies highlight the value of advanced techniques for overcoming the limitations imposed by the material characteristics of carbonized papyri, opening new possibilities for deciphering and publishing their contents. Among the imaging methods, Pulsed Thermography (PT) has emerged as a promising technique for the non-invasive analysis of complex and fragile cultural heritage^20–24^. The working principle is based on the sample stimulation with a pulse of visible light and the recording of the consequent emitted infrared radiation variation. The technique can be successfully applied to both optically opaque and optically semi-transparent materials: in the former case, such as for bronze statues, both the VIS light absorption and the IR emission recorded by the camera take place at the sample surface level, being the PT signal proportional to the temperature variation at the surface itself. In this case, the thermal contrast depends on subsurface local inhomogeneities in the thermal properties associated, for instance, with the presence of cracks or voids^25–27^. For optically semi-transparent materials, i.e. paper-based artifacts such as papyri, the PT signal recorded by the camera is contributed by a weighted average of the IR emission within the sample thickness, whose limited range (2–3 °C) cannot be considered harmful for the samples. PT has displayed a wide range of applications in Heritage Science for detecting hidden features in layered structures, such as graphic elements, defects or material heterogeneities in paintings^28–32^, frescoes^33–36^, and manuscripts^37–39^, as well as other various materials^43–48^. So far, its potential for analyzing carbonized papyri, such as the Herculaneum scrolls, and papyri in general, had remained unexplored. This study documents for the first time the application of PT to the analysis of ancient papyri with the main purpose of improving textual legibility and investigating structural features. By setting the proper measurement frequencies, textual detection was possible from the analysis of short-time thermal sequences acquired at the maximum frame rate and spatial resolution. A lower frame rate was adopted for capturing the longer heat propagation within the papyrus substrate for morphological examination. Through advanced data analysis techniques, such as contrast enhancement algorithms, Thermal Recovery (TR) mapping, and Pulsed Phase Thermography (PPT), the objectives of this research were achieved: unreadable text was revealed, the legibility of partially visible text was enhanced, and both structural and morphological insights were obtained, including fibers patterns and areas of adhesion of the papyrus substrate to the paperboard. The present study proves the potential of this technique for the investigation of papyri not only as far as textual recovery, but also as far as structural analysis is concerned.

## Materials and methods

### Samples description

Each fragment, whose thickness is about 0.15 mm, is glued to a paperboard, which is slightly thicker than the papyrus sheet (ca. 0.30 mm), and is itself fastened to a piece of cardboard or a wooden tablet, which is about 50 mm thick. Owing to their fragility and critical conservative conditions, papyrus fragments cannot be removed from this support nor placed vertically for experimental analyses. All fragments are distributed across metallic frames (‘cornici’), which are kept in the Officina dei Papiri Ercolanesi of the National Library ‘Vittorio Emanuele III’ of Naples. The text is written in vertical columns slightly slanting to the left of uniform size within the same roll, but different from roll to roll, with individual letters measuring around 3–4 mm.

The following four papyri were selected for analysis:

1. *PHerc*. 1018 (end of first century BC/beginning of first century AD) was unrolled in 1808 and holds Philodemus of Gadara’s *History of the Stoa*. It consists of twenty-one papyrus fragments stored in twelve ‘cornici’, of which the fragments analyzed by us (‘cornici’ 1 and 2) measure 17 cm in height and 25.6 cm in length, and 19 cm in height and 27.5 cm in length, respectively^49^.
2. *PHerc.* 1691/1021 (second quarter of first century BC) was mechanically unrolled between 1782 and 1795 and contains a graphically inaccurate and textually provisional version of Philodemus’ *History of the Academy*. It consists of nine papyrus fragments, contained in nine ‘cornici’, of which the fragment analyzed by us (*PHerc.* 1021, ‘cornice’ 2) measures 20.6 cm in height and 36.9 cm in length^50^.
3. *P.Herc*. 1780 (latter half of first century BC) was unrolled in 1852 and hands down the so-called *History of the Garden* by, possibly, Philodemus. It consists of eight papyrus fragments stored in eight ‘cornici’, of which the fragment analyzed by us (‘cornice’ 7) measures 19.2 cm in height and 34.2 cm in length^51^.
4. *PHerc.* 1025 (first century AD) was unrolled between 1802 and 1803 and transmits an anonymous Epicurean ethical treatise by, possibly, again Philodemus. It consists of twenty-seven papyrus fragments stored in fifteen ‘cornici’, of which the fragment analyzed by us (‘cornice’ 1) measures 14.6 cm in height and 29.7 cm in length^52^.

### Pulsed thermography measurements

The PT measurements were performed using a FLIR X6800sc infrared camera (MWIR spectral range 3.5–5 μm, cooled FLIR InSb detector, 25 μm pitch, FPA 640 × 512 pixels, frame rate 520 Hz at the maximum spatial resolution, NETD < 20 mK at 30 °C) positioned perpendicularly to the investigated papyrus. A sample heating of ca. 2–3 °C was produced by two flash lamps (maximum power of 3 kW) powered by a Hensel (EH Mini speed flash head and Tria 24 generators), and oriented at 45° with respect to the papyrus surface, as reported in the scheme in Figure S3. The flash lamps were equipped with custom glass&water filters in order to avoid exposing the sample to UV radiation, that could trigger photodegradation, and to prevent IR radiation from the heated lamps from reaching the papyrus surface and be reflected toward the camera, so negatively affecting the contrast of the ink appearing in the thermograms. Pulsed thermography in reflection modality was used to ensure that the method was completely non-destructive, as the samples cannot be detached from their cardboard or wooden support without suffering irreversible damage. For these reasons, transmitted or backlighting thermography was not considered as an experimental approach.

Two series of acquisitions were carried out depending on the particular feature investigated. The writing, which is located on the front surface of the papyrus, is expected to appear with the maximum contrast in the very first thermograms after the flash pulse. Accordingly, the maximum possible frame rate (520 Hz) has been used, keeping the spatial resolution of the camera at its maximum value. Thus, only a few thermograms have been recorded after the flash pulse not to have too heavy files to be elaborated in the post-processing phase. On the other hand, in order to analyze the papyrus structure and morphology, a much lesser frame rate (128 Hz) was instead used. In this case, longer measurements have been carried out letting the heat generated after pulse light absorption to reach the bottom of the papyrus.

FLIR Research Studio 3.1.0 software was used to manage the acquisition process and extract single images from the thermographic sequences. Image elaborations for textual analysis comprehended contrast enhancements by means of Matlab algorithms and GIMP sharpen masks and the final image stitching by using ControlPoints function in PTgui and ICE software.

For morphological evaluations, TR maps were calculated following a methodology similar to that used in previous studies^35,42^ The maximum induced temperature gradient was first computed for each pixel in the thermal frames using Eq. (1).

1 $$\Delta T\left( {i,j} \right)={T_{max}}\left( {i,j} \right) - {T_0}\left( {i,j} \right)$$

where *T*_*0*_ is the temperature of the pixel at coordinates *(i*,* j)* in the frame captured immediately before the external heating begins (flash stimuli), and *T*_*max*_ is the temperature of the same pixel immediately after the heating ends. Subsequently, the temporal evolution of each pixel was analyzed to estimate the time required to recover 50% of its respective induced *ΔT*. According to this analysis, this threshold provides the most significant variation in recovery times across the map pixels. However, varying the threshold within 45–55% does not significantly affect the overall results.

For what concerns PPT analysis, it allows for the analysis of the material’s thermal response in the frequency domain. This is achieved by applying a discrete one-dimensional Fourier transformation to each pixel in the sequence of thermographic images^53–56^. Amplitude and phase maps are generated by applying the calculation across the sequence.

Given a total of *N* thermograms in the dataset, the number of distinct frequency values obtainable is *N/2*, due to the symmetry of the discrete Fourier transform. The corresponding discrete frequencies *f*_n_ are defined as Eq. (2):

2 $${f_n}=n/(N \times \Delta t)$$

where *Δt* is the time interval between consecutive thermal images, and *n* ranges from 0 to *N/2*.

Amplitude images affected by the inhomogeneity of the thermal illumination, did not provide significant analytical insights and were therefore discarded. The phase images that most effectively highlighted the structural features of the papyrus and were used for our analysis were those obtained at a frequency of 0.2 Hz. The first phase images acquired at 0.1 Hz show saturation effects, while the images corresponding to higher frequencies (above 0.3 Hz) exhibit a contrast comparable to or lower than that observed at 0.2 Hz. TR analysis, as well as PPT images, were calculated using custom MATLAB (R2019b, Math-Works) scripts.

It is worth noting that the small and transient heating induced by the PT measurements (2–3 °C) does not induce an energy variation sufficient to cause damage. Indeed, it is far below any threshold that could cause chemical or mechanical modification of the papyrus fibers, which have undergone carbonisation by thermal shock at 320 °C during the eruption of Mount Vesuvius in 79 AD^3,4^. Moreover, the thermal increase is extremely localized in time (few milliseconds), and the sample rapidly returns to equilibrium with the ambient conditions, preventing any cumulative or long-lasting effects.

## Results

Thermal imaging successfully revealed textual portions almost illegible to the naked eye and enhanced only partially visible ones through NIR technical photography, that is here used as a comparison method^46^. Secondly, structural and morphological insights were addressed, such as fibers patterns and adhesion points to the paperboard, which provided valuable information about former conservative techniques and the current state of conservation. PT measurements were carried out by stimulating the selected papyri with two flash lamps (3 kW) and recording the emitted IR radiation from the sample by an IR camera. Each papyrus fragment was virtually divided into multiple inspection areas (framing), measuring approximately 15 cm × 12 cm, to partially overlap with adjacent areas and ensure continuity of information. Finally, a reconstructed map of each papyrus fragment was generated by mosaicking the single inspection areas, called thermograms.

### Textual enhancement

By applying PT to different papyrus fragments, the legibility of the Greek text contained in them was significantly enhanced in comparison with NIR technical photography, owing to the excellent contrast between ink and papyrus substrate achieved. Single frames were extracted where the contrast allowed the detection of Greek text from the papyrus substrate, typically in correspondence with the first frame recorded after the flash pulse. Since the presence of thin graphic features is not expected to significantly affect the local heat diffusion in the inspected material, the thermogram contrast in the PT investigation of written semi-transparent artefacts is likely to be originated from local difference in both the VIS absorption and the IR emission properties of the ink with respect to the papyrus substrate^47,48,57^. This experimental evidence may be justified by the occurrence that the ink located on the visible surface of the specimen has a larger absorption coefficient in the visible range than the surrounding material, i.e. the charred papyrus substrate. Accordingly, when the light pulse reaches the sample surface and is absorbed and converted into heat, the inked areas have an increased temperature with respect to the surrounding layer and, thus, a consequent increase in emitted infrared radiation. Because of this phenomenon, the Greek text shows up with an excellent contrast in the thermograms recorded just after the light pulse.

**Fig. 1:**
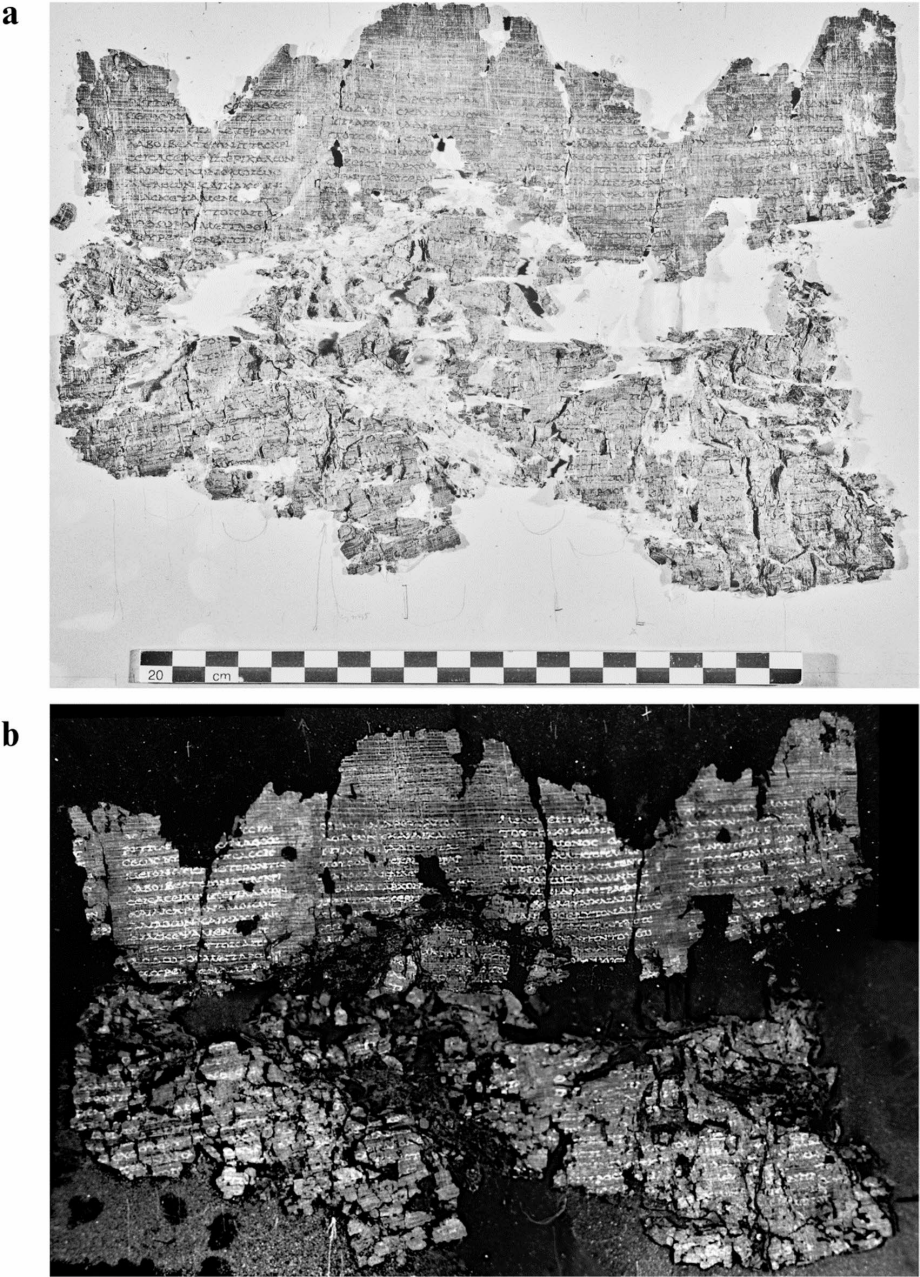
*P. Herc.* 1018, ‘cornice’ 1: (**a**) NIR image at 1000 nm acquired through technical photography. By permission of Ministero della Cultura (photocredit: Biblioteca Nazionale “Vittorio Emanuele III,” Napoli–Consiglio Nazionale delle Ricerche, Istituto di Scienze del Patrimonio Culturale); (**b**) thermographic mosaic showing the enhancement of the Greek text. By permission of Ministero della Cultura (photocredit: Biblioteca Nazionale “Vittorio Emanuele III,” Napoli–Consiglio Nazionale delle Ricerche, Istituto di Scienze del Patrimonio Culturale).

Figure 1 shows the promising results obtained for *P.Herc.* 1018, ‘cornice’ 1, belonging to Philodemus’ *History of the Stoa*^49^. The thermographic reconstruction (Fig. 1b) is compared with the corresponding NIR image at 1000 nm^44^ (Fig. 1a) in order to evaluate the quality of the textual improvement, both in terms of layout and letters detection. In particular, an optimal reading of the top of four text columns is offered by the thermographic mosaic, while several letters were better revealed in the lower part of the papyrus fragment, which appears to be morphologically much more complex and multilayered.

A concrete example is offered, among many others, by lines 4–5 of column 3: the thermographic image confirms, without any doubt, the new reading προεµνήϲ|˹θ͙η͙˺µ̣ε̣ν̣, ‘we mentioned earlier’, recently advanced by scholars^58^, instead of the previous one προεµνήϲ|α̣µ̣ε̣ν̣ ^59,60^.

**Fig. 2:**
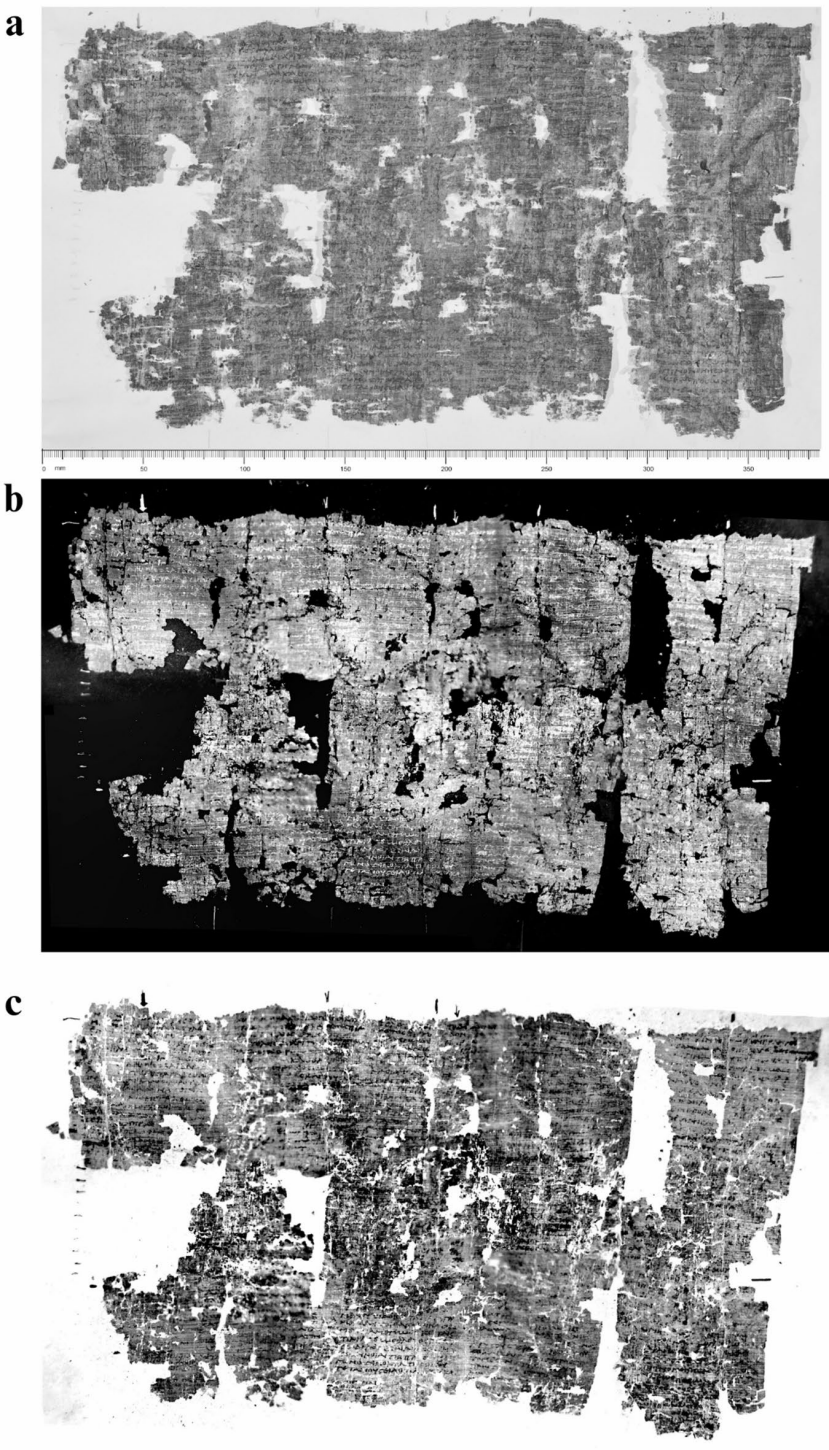
*P. Herc.* 1021, ‘cornice’ 2: (**a**) NIR image at 1000 nm acquired through technical photography, in which the Greek text is hardly visible; (**b**, **c**) thermographic mosaic displaying the enhanced text through standard and inverted palette, respectively. By permission of Ministero della Cultura (photocredit: Biblioteca Nazionale “Vittorio Emanuele III,” Napoli–Consiglio Nazionale delle Ricerche, Istituto di Scienze del Patrimonio Culturale).

In Fig. 2, a comparison between the NIR image at 1000 nm and PT reconstructions, visualized with a different color palette, is reported for *P.Herc.* 1021, ‘cornice’ 2, belonging to Philodemus’ *History of the Academy*^50^, a papyrus characterized by a very dark substrate (see Figure S1) and barely legible even through NIR technical photography^46^ (Fig. 2a). Also in this case, the full mosaic was obtained from the extraction of the first frames of the thermographic sequence. In this way, the remnants of five, though partially lacunose, text columns were better revealed offering a full glimpse of written and unwritten parts and a good detail of single letters. The degree of textual readability was assessed by also evaluating the efficacy of the specific thermal palette adopted in the image for the textual visualization (Fig. 2b, c).

Among the many possible instances to be mentioned, a good example is represented by lines 34–37 of column 9: the new reconstruction of the text contained in the last edition of this bookroll^50^ is now validated by the thermographic image, given the physical continuity of the papyrus and the exclusion of ancient drawings (‘disegni’) from the reconstruction: ⌈ἐ͙⌉|[ϲτ]ε̣φάν̣⌈ο⌉υν τ̣⌈ὸ͙⌉ν [Ἡρ]ακλεί|[δη]ν παρ⌈ε⌉ϲτηκότ̣α τ̣⌈ῶ͙⌉ι̣ κή|[ρυκ]ι, ‘they honoured with a crown Heraclides, who was standing next to the herald’.

**Fig. 3:**
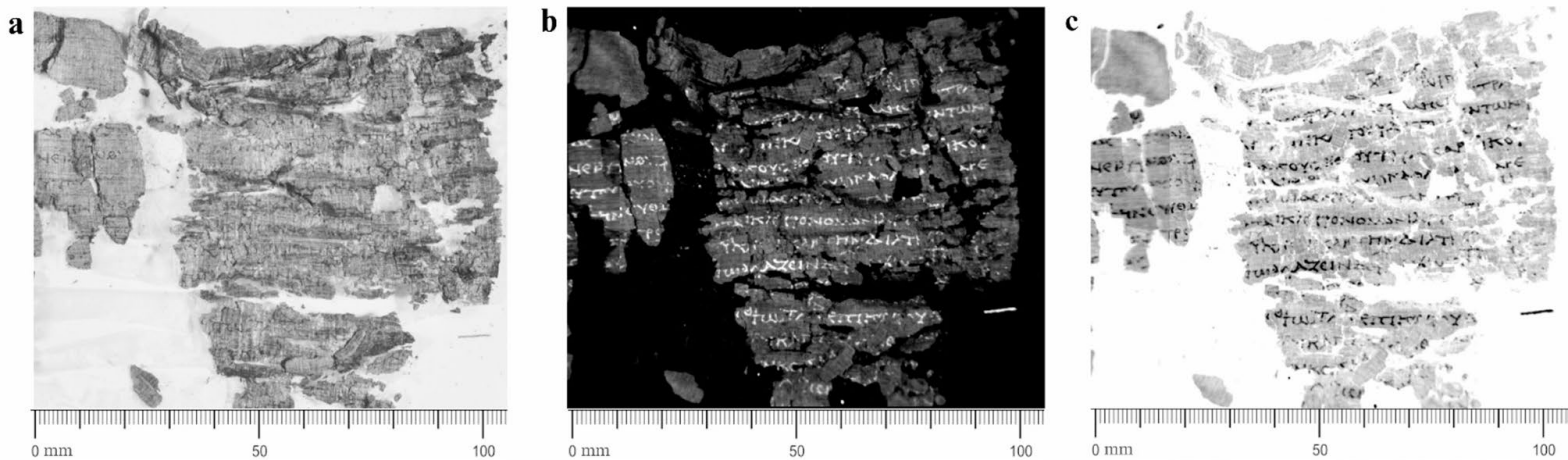
*P.Herc* 1780, ‘cornice’ 7, detail: (**a**) NIR image acquired at 1000 nm, in which the text is hardly visible. By permission of Ministero della Cultura (photocredit: Biblioteca Nazionale “Vittorio Emanuele III,” Napoli–Consiglio Nazionale delle Ricerche, Istituto di Scienze del Patrimonio Culturale); (**b**, **c**) thermographic images showing optimal textual enhancement through standard and inverted palette, respectively. By permission of Ministero della Cultura (photocredit: Biblioteca Nazionale “Vittorio Emanuele III,” Napoli–Consiglio Nazionale delle Ricerche, Istituto di Scienze del Patrimonio Culturale).

Finally, Fig. 3 shows the results obtained for *P.Herc.* 1780, ‘cornice’ 7, belonging to the so-called *History of the Garden* by, possibly, again Philodemus^61^. In this case, the not-planar morphology and the severe multi-layering of the papyrus substrate make the text hardly legible even in the NIR image at 1000 nm (Fig. 3a). On the contrary, the thermographic results provide the best contrast and readability ever attained for this papyrus, which is one of the darkest and most inaccessible of the papyrus collection. Fig. 3b, c show clearly the advantages offered by the comparison of both black and white palettes for the textual recovery.

It is important to note that single letters may appear distorted owing to the different surface morphology of the selected papyri. This irregularity complicates reading through optical systems, as achieving an optimal focus requires adjustments for individual textual areas. Nevertheless, the presented approach demonstrates that the PT images allow the identification of text located on different planes, albeit with varying degrees of readability.

### Structural and morphological analysis

The application of PT to the same papyrus fragments also provided valuable information about their structural and morphological features, such as the fibers texture and adhesion points to paperboard. The thermal frames recorded after stimulation were analyzed using two different processing methods: thermal recovery (TR) mapping^35,42^ and pulsed phase thermography (PPT)^62,63^.

**Fig. 4:**
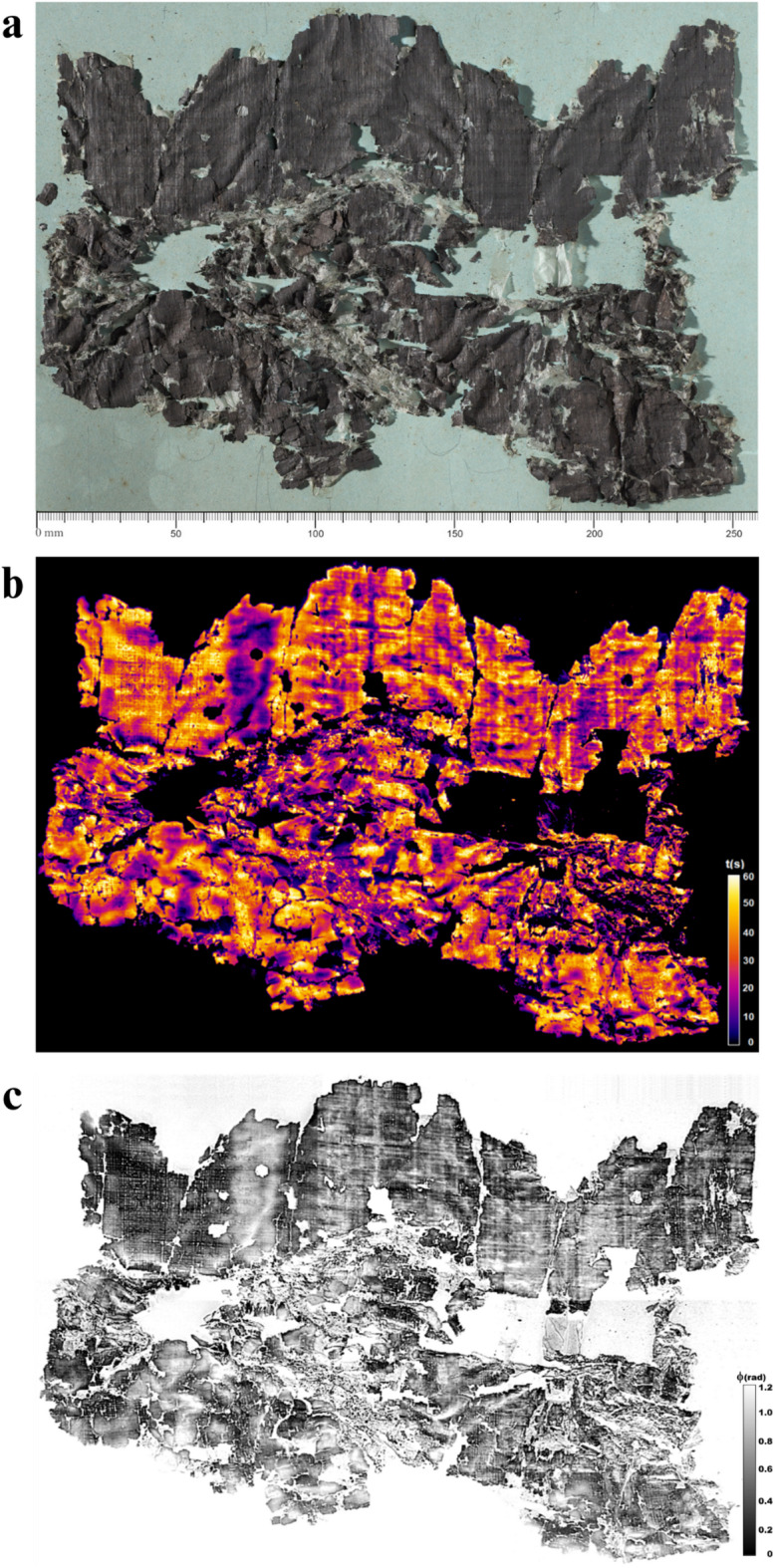
*P. Herc* 1018, ‘cornice’ 1: (**a**) visible image acquired with raking light; (**b**) TR map showing regions with different recovery times probably due to variations in height of the papyrus with respect to the paperboard; (**c**) phase image obtained by PPT at 0.2 Hz, where both fiber pattern and whitish ‘clouds’ are displayed. By permission of Ministero della Cultura (photocredit: Biblioteca Nazionale “Vittorio Emanuele III,” Napoli–Consiglio Nazionale delle Ricerche, Istituto di Scienze Applicate e Sistemi Intelligenti).

In the TR map of *P.Herc.* 1018, ‘cornice’ 1 (Fig. 4b), from a thermophysical perspective, regions with lower recovery times (in violet or purple) correspond to areas in direct contact with the paperboard, which acts as a heat sink, accelerating heat dissipation, or characterized by lesser depth, as arises from the comparison with the corresponding visible image acquired with raking light^44^ (Fig. 4a). Conversely, regions with higher recovery times (in red or yellow) identify areas where the papyrus is not in direct contact with the support because of its greater depth. In these cases, the air between the papyrus and the paperboard serves as an insulator, slowing heat dissipation and increasing recovery times. Notably, contact regions are partly influenced by the presence of gluing points on the paperboard. This information regarding the adhesion regions is particularly valuable for restoration efforts, helping conservators determine intervention strategies for the preservation of these fragile manuscripts. Regions with different recovery times may also be ascribable to variations in thickness due to either glueing areas between adjacent sheets (*kolleseis*) or overlapping layers, i.e. over- and underlying layers which have remained stacked to the ground layer during the unrolling process through the Piaggio machine. For the PPT analysis, phase images were calculated and reconstructed from the recorded thermal frames^62,63^. In general, PPT allows the calculation of amplitude and phase images at different frequencies corresponding to progressively deeper layers of the papyrus substrate. Phase images provided the most relevant information, highlighting structural and morphological features of the papyrus, such as fiber patterns and surface corrugation (Fig. 4c). Here, however, the fiber pattern appears blurred or completely obscured in other regions by whitish ‘clouds’ that roughly correspond to the areas in which the papyrus adheres to the paperboard, as indicated by violet/purple colors in the corresponding TR maps.

**Fig. 5:**
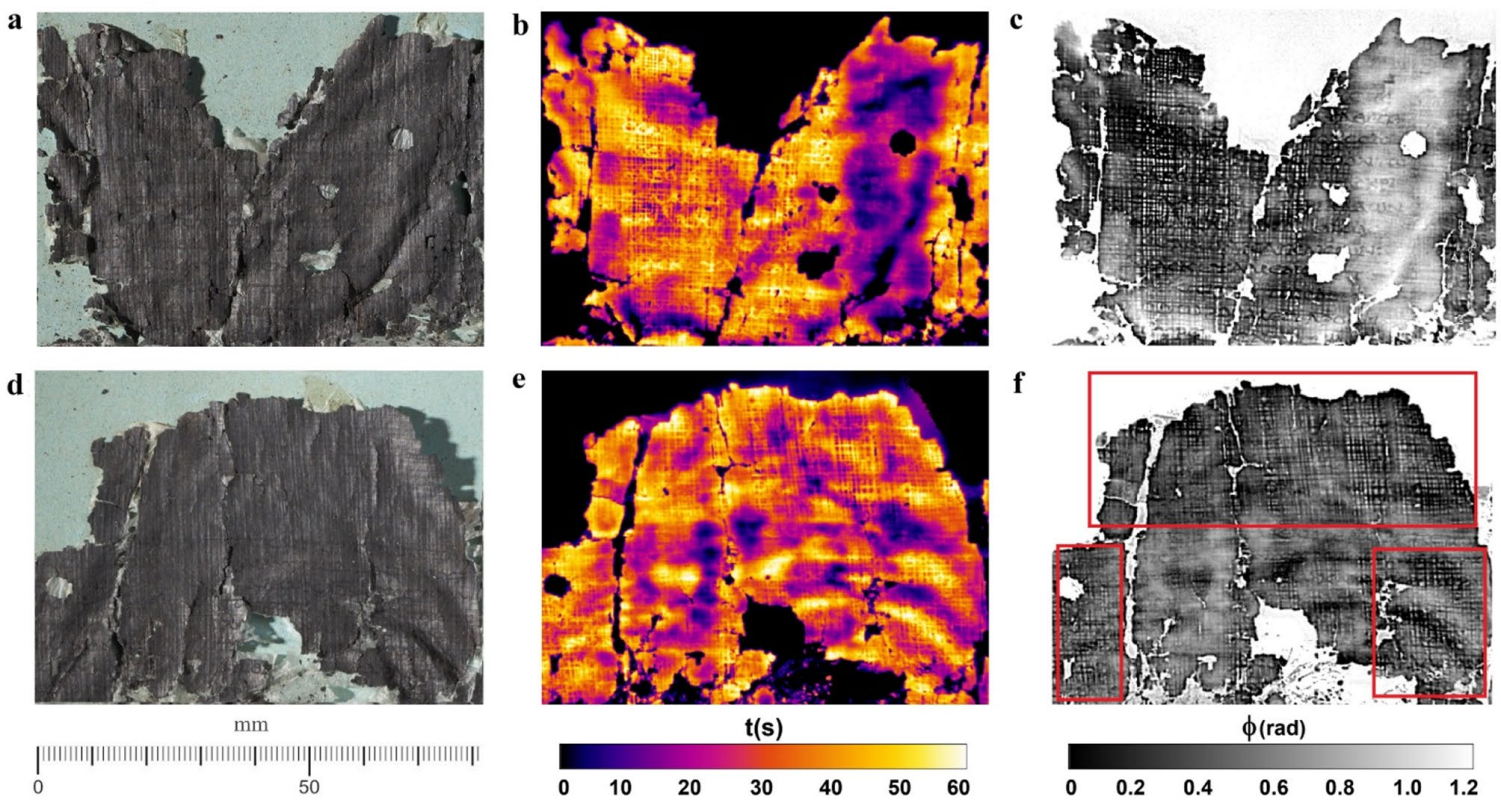
*P.Herc* 1018, ‘cornice’ 1: (**a**–**c**) raking-visible light, TR mapping, and phase image obtained by PPT of selected regions of the papyrus fragment; *P.Herc* 1018, ‘cornice’ 2: (**d**–**f**) raking-visible light, TR mapping, and phase image obtained by PPT of selected regions of the papyrus fragment, where the fibers pattern is clearly visible (squared-marked areas in (**f**)). By permission of Ministero della Cultura (photocredit: Biblioteca Nazionale “Vittorio Emanuele III,” Napoli–Consiglio Nazionale delle Ricerche, Istituto di Scienze Applicate e Sistemi Intelligenti).

Figure 5 shows details of TR mapping and PPT elaborations on selected regions from *P.Herc*. 1018, ‘cornice’ 1 (Fig. 5b-c) and ‘cornice’ 2 (Fig. 5e-f) compared with visible images acquired using raking light (Fig. 5a, d, respectively). In Fig. 5c, f the papyrus fiber weave is clearly visible, particularly across the entire area in Fig. 5c and in the regions squared in red in Fig. 5f. Here, the whitish ‘clouds’ are visible as well.

For another example of this comparison (*P.Herc.* 1025, ‘cornice’ 1) see Figure S2. So, as it appears, the blurring can mostly be attributed to the corrugation of the papyrus substrate and its consequent variations in depth with respect to the paperboard. These findings demonstrate the potential of thermographic techniques to reveal not only textual but also structural and morphological details.

## Discussion

The investigation of Herculaneum papyri remains a remarkable challenge for scientists today owing to their state of carbonization and the difficulty of differentiating the carbon-based ink from the darkened papyrus substrate. While advanced imaging techniques developed in recent decades have enhanced the ability to read and interpret these manuscripts, the challenge of simultaneously obtaining both textual and structural information in a non-invasive manner had remained an unexplored issue. This study introduces PT as a novel and highly promising technique for the non-invasive examination of these ancient manuscripts in the mid-wave infrared range. The limited range of the light stimulus and the consequent low variation in temperature induced within the samples (2–3 °C) cannot be regarded as harmful for ancient manuscripts or papyri. Far the less so as far as carbonized papyri are concerned, such as the Herculaneum scrolls, which – as mentioned – underwent a thermal shock at ca. 320 °C for a few seconds as a consequence of the volcanic surge that buried Herculaneum in 79 AD. By leveraging thermal imaging and advanced data processing techniques, PT effectively enhances the contrast between ink and papyrus substrate, revealing text either invisible o hardly visible to the naked eye and enhancing the legibility of partially readable one. Additionally, it helps identifying adhesion areas and fibers pattern enabling the characterization of the substrate’s morphology, which is also useful for conservation efforts. Although previous studies employing different spectral regions (particularly in the shortwave-infrared range) have demonstrated the capability to recover text with comparable or even higher contrast than that achieved in this work^18^, PT presents the distinct advantage of simultaneously facilitating both textual recovery and structural analysis within a single methodological framework. While parameter optimization remains necessary for different papyri owing to variations in degradation states, our findings highlight the significant potential of thermographic techniques for the study and preservation of carbonized papyri. Future research should explore the integration of PT with other diagnostic techniques to further refine its capabilities and expand its applicability to a broader number of samples. By enabling the simultaneous acquisition of multiple types of information, it may serve as a valuable and complementary tool to other techniques, contributing to the long-term preservation of Herculaneum papyri. Moreover, the readability of the text extracted through PT could be further improved by employing pattern recognition and artificial intelligence-based analysis techniques, which may enhance the differentiation between ink and papyrus substrate and facilitate textual edition and interpretation of these unique manuscripts.

## Supplementary Information

Below is the link to the electronic supplementary material.

Supplementary Material 1

## Data Availability

All data needed to evaluate the conclusions in the paper are present in the manuscript and in the Supplementary Information. Additional data available from authors upon request.
