# Supplementary material for: Pulsed thermographic analysis of Herculaneum papyri
Source: Sci Rep. 2025 Oct 8;15:34466. doi: 10.1038/s41598-025-19911-w (PMC12508051; doi:10.1038/s41598-025-19911-w)
Supplement: Supplementary file 1 — Supplementary Material 1 [file 41598_2025_19911_MOESM1_ESM.docx]

**Supplementary Information for**

***Pulsed thermographic analysis of Herculaneum papyri***

Sofia Ceccarelli, Massimo Rippa, Giovanni Caruso, Simona Boccuti, Melania Paturzo, Vito Pagliarulo, Kilian Fleischer, Loredana Luvidi, Costanza Miliani^*^, Graziano Ranocchia^*^

*Corresponding authors: Costanza Miliani, Graziano Ranocchia

Email: [costanza.miliani@cnr.it](mailto:costanza.miliani@cnr.it); [graziano.ranocchia@unipi.it](mailto:graziano.ranocchia@unipi.it)

**This PDF file includes:**

Fig. S1 – *PHerc*.1021, ‘cornice’ 2: VIS image acquired with raking light.

Fig. S2 – *PHerc*.1025, ‘cornice’ 1: VIS image acquired with raking light, thermographic mosaic, TR and PPT maps.

Fig. S3 – Thermographic setup.

**
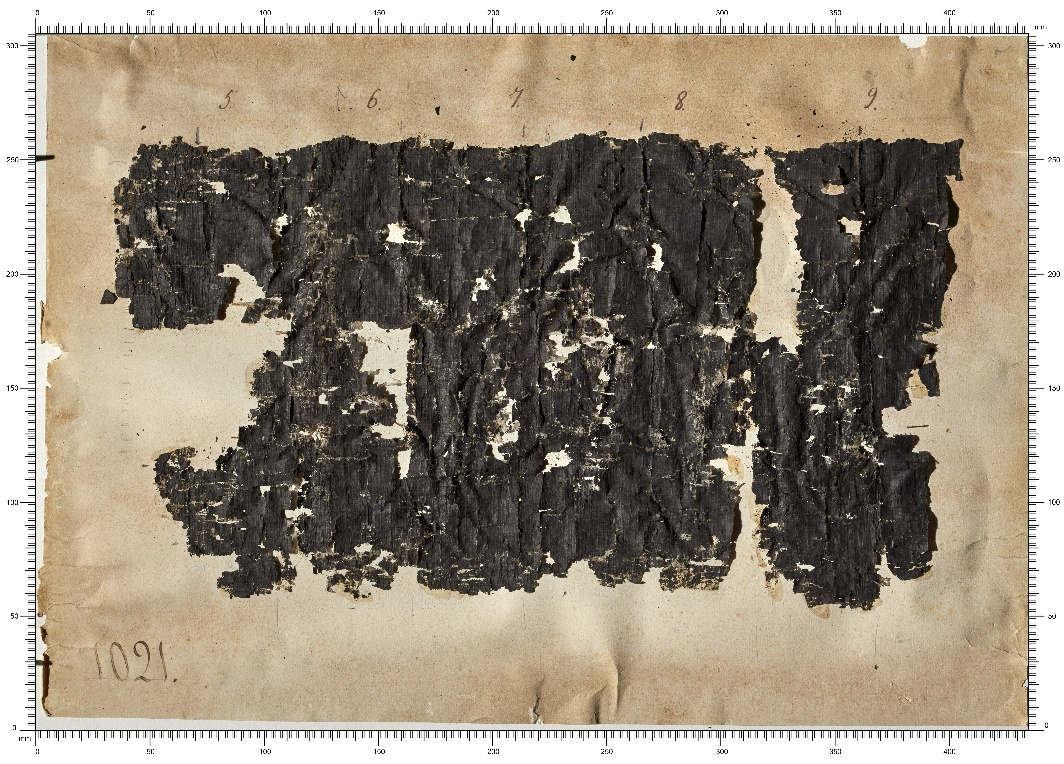
**

**Figure S1.** *PHerc*.1021, ‘cornice’ 2: VIS image acquired with raking light. The high level of carbonization of the papyrus substrate does not reveal the Greek text while highlighting the irregular morphology of the papyrus substrate.


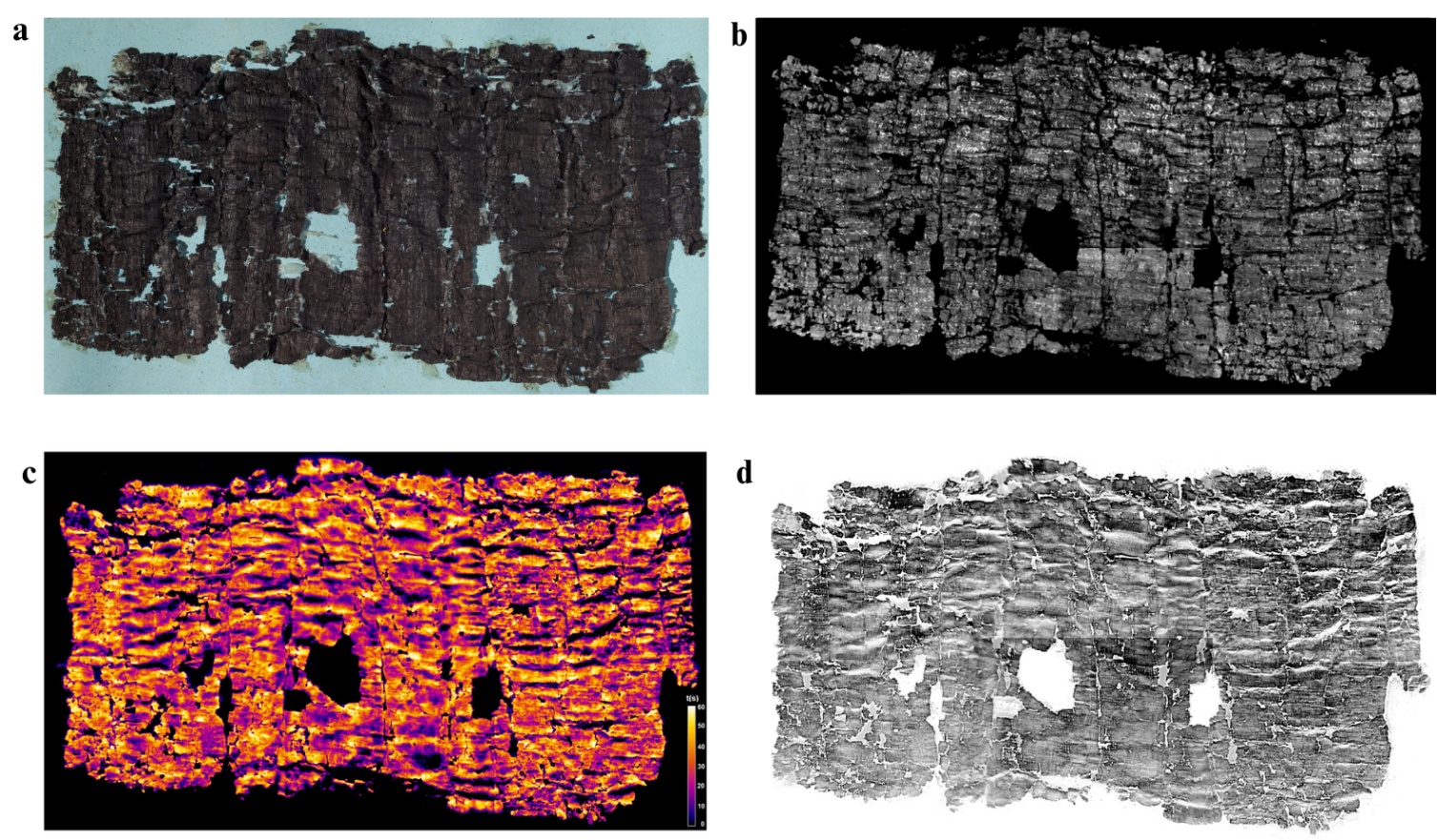


**Figure S2.** *PHerc*.1025, ‘cornice’ 1: a) VIS image acquired with raking light, b) thermographic mosaic, c) TR map, d) PPT map at 0.2 Hz.

**
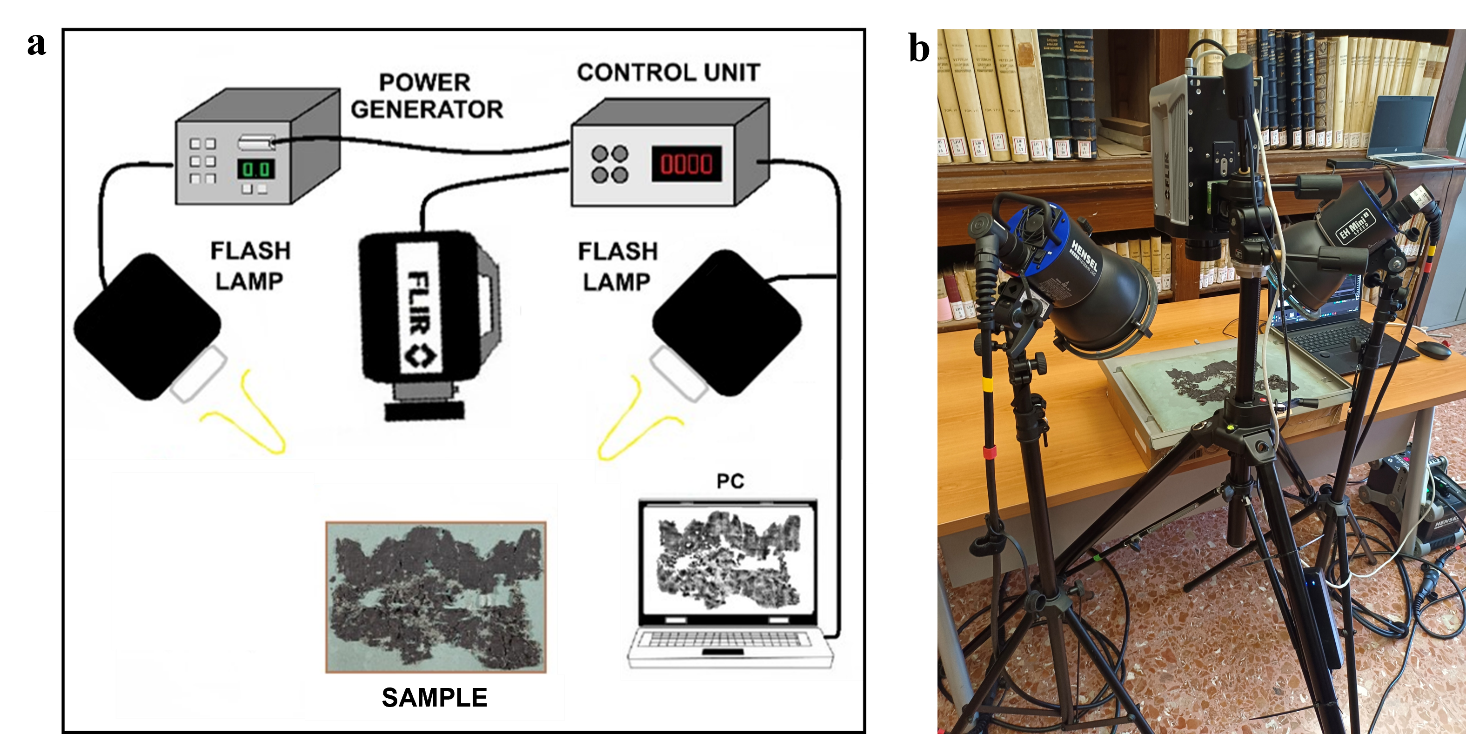
**

**Figure S3.** Thermographic setup: a) schematic representation of the PT system components; b) picture of the acquisition arrangement.
